# Clinical periodontal indices are associated with systemic inflammatory and oxidative stress biomarkers in dogs with periodontal disease

**DOI:** 10.1016/j.vas.2026.100882

**Published:** 2026-09-24

**Authors:** Negin Sharei, Nooshin Derakhshandeh, Milad Faraji, Artin Sheibani, Saeed Nazifi

**Affiliations:** a Department of Clinical Sciences, School of Veterinary Medicine, Shiraz University, Shiraz, 71441-11731, Iran; b Department of Basic Sciences, School of Veterinary Medicine, Shiraz University, Shiraz, Iran; c Department of Pathobiology, School of Veterinary Medicine, Shiraz University, Shiraz, Iran

**Keywords:** Canine periodontal disease, C-reactive protein, Serum amyloid A, Plaque index, Gingival index, Systemic inflammation

## Abstract

**Background:**

Periodontal disease is a common chronic inflammatory condition in dogs and may be associated with systemic inflammatory and oxidative alterations. However, the relationship between clinical periodontal severity and circulating inflammatory and redox biomarkers remains insufficiently characterized.

**Objectives:**

This study aimed to evaluate the associations between periodontal status, clinical periodontal indices, and systemic inflammatory and oxidative stress biomarkers in client-owned dogs.

**Methods:**

A total of 131 client-owned dogs were enrolled in this cross-sectional observational study, including 70 clinically healthy dogs and 61 dogs with periodontal disease. Periodontal status was assessed using visual evaluation of plaque index (PI) and gingival index (GI). Serum concentrations of interleukin-6 (IL-6), C-reactive protein (CRP), serum amyloid A (SAA), malondialdehyde (MDA), and total antioxidant capacity (TAC) were measured. Group comparisons and correlations between periodontal indices and biomarkers were performed using non-parametric statistical analyses.

**Results:**

Dogs with periodontal disease showed significantly higher serum concentrations of IL-6, CRP, SAA, and MDA, along with reduced TAC levels, compared with healthy dogs (*p* < 0.05). Increasing PI and GI severity categories were associated with progressive alterations in inflammatory and oxidative biomarkers. PI demonstrated very strong positive correlations with CRP, SAA, and IL-6, whereas GI showed significant but comparatively weaker correlations with these inflammatory markers. MDA showed moderate positive associations, while TAC exhibited weak inverse correlations with periodontal indices. After adjustment for demographic confounders, periodontal disease status remained significantly associated with increased IL-6, CRP, SAA, and MDA concentrations and decreased TAC levels. Age showed modest associations with periodontal indices, whereas sex was not significantly related to periodontal severity or biomarker concentrations.

**Conclusions:**

Canine periodontal disease is associated with coordinated systemic inflammatory activation and oxidative imbalance. The stronger associations observed for inflammatory biomarkers compared with oxidative stress markers suggest that systemic inflammation represents the predominant biological response accompanying disease progression. Integrated evaluation of clinical periodontal parameters and circulating biomarkers may provide additional insight into the biological characteristics of canine periodontal disease, although longitudinal studies are required to determine causal relationships.

## Highlights

• Canine periodontal disease was associated with systemic inflammatory changes.
• IL-6, CRP, and SAA strongly correlated with periodontal severity indices.
• Periodontal disease was linked to oxidative imbalance with reduced TAC levels.
• Plaque accumulation showed stronger biomarker associations than gingival inflammation.
• Multivariable models confirmed biomarker alterations after confounder adjustment.

## 1 Introduction

Periodontal disease is one of the most prevalent chronic inflammatory conditions affecting companion animals, particularly dogs, and remains a major cause of oral discomfort, tooth loss, and reduced welfare (Hendy et al., 2026). The disease encompasses a progressive pathological continuum that ranges from reversible gingival inflammation to irreversible periodontitis, characterized by the destruction of periodontal ligament fibers, alveolar bone resorption, gingival recession, and eventual tooth instability and loss (Dubey, 2020). Dental plaque biofilm is widely recognized as the primary initiating factor; however, disease onset and progression are strongly modulated by host immune responses, microbial dysbiosis, and systemic physiological factors, including metabolic status, endocrine regulation, oxidative balance, and environmental influences (Dubey, 2020; Wallis & Holcombe, 2020).

Beyond local periodontal tissue damage, persistent microbial stimulation within diseased periodontal tissues can promote sustained host immune activation and the release of inflammatory mediators into the systemic circulation (Hajishengallis & Chavakis, 2021). This provides a biological rationale for investigating the relationship between periodontal disease and systemic inflammatory alterations and has increased interest in circulating biomarkers as potential indicators of periodontal-associated systemic burden (Bhuyan et al., 2022; Harvey, 2022). Among the mediators involved in this inflammatory response, interleukin-6 (IL-6) plays a central role in coordinating innate and adaptive immune responses and stimulating hepatic acute-phase protein synthesis. C-reactive protein (CRP) and serum amyloid A (SAA) are important acute-phase proteins that can reflect systemic inflammatory activity and may respond dynamically to inflammatory stimuli (Papareddy & Herwald, 2025; Sabine Elisabeth et al., 2025).

Periodontal inflammation is also closely linked to redox imbalance. Persistent recruitment and activation of inflammatory cells can increase reactive oxygen species (ROS) generation, while prolonged oxidative challenge may overwhelm endogenous antioxidant defenses and promote lipid, protein, and nucleic acid damage. Malondialdehyde (MDA), a product of lipid peroxidation, is therefore commonly used as an indicator of oxidative injury, whereas total antioxidant capacity (TAC) provides an integrated estimate of the capacity of circulating antioxidant systems to counteract oxidative stress (Mohideen et al., 2023; Patil et al., 2024). Assessment of inflammatory and oxidative biomarkers together may consequently provide a broader representation of the systemic biological response associated with periodontal disease than either domain evaluated independently.

Clinically, periodontal status can be characterized using indices such as the plaque index (PI) and gingival index (GI), which provide standardized measures of plaque accumulation and gingival inflammation, respectively (Metin et al., 2023). These indices represent biologically related but distinct components of periodontal pathology: PI reflects the local microbial burden, whereas GI primarily reflects the host inflammatory response. Consequently, examining their relationships with circulating biomarkers may help determine whether systemic biochemical alterations are more closely associated with microbial accumulation, local tissue inflammation, or both. Although plaque accumulation is considered a principal initiating factor in periodontal disease (Abdulkareem et al., 2023), the extent to which PI and GI differ in their associations with systemic inflammatory and oxidative biomarkers in dogs remains insufficiently characterized.

Previous canine studies have reported alterations in inflammatory and oxidative parameters in association with periodontal disease; however, the available evidence remains fragmented, with investigations frequently focusing on individual inflammatory or oxidative markers rather than evaluating these biological domains concurrently in relation to standardized clinical periodontal indices (Moradi Haghgoo et al., 2023; Rodean et al., 2025; Valiyaveetil Karattil et al., 2022). Furthermore, the strength and consistency of associations between PI or GI and circulating biomarkers have not been comprehensively compared across inflammatory and oxidative pathways. This is particularly relevant for SAA, a highly responsive acute-phase protein whose relationship with the severity of naturally occurring periodontal disease in dogs remains comparatively underexplored (Christensen et al., 2013; Oikonomidis et al., 2025).

Another unresolved issue is whether demographic characteristics modify the relationship between periodontal status and systemic biochemical responses. Aging is associated with greater periodontal disease burden in dogs, but its contribution to circulating inflammatory and oxidative profiles in the context of periodontal disease remains insufficiently defined. Similarly, evidence for sex-related differences in canine periodontal status and associated systemic biomarkers remains limited (O’Neill et al., 2021; Shiau & Reynolds, 2010). These factors are therefore relevant when interpreting systemic biomarker alterations in naturally occurring periodontal disease.

Taken together, the principal knowledge gap is not simply whether individual inflammatory or oxidative biomarkers are altered in dogs with periodontal disease, but whether clinical measures of plaque accumulation and gingival inflammation show differential and coordinated relationships with systemic inflammatory and redox biomarkers. Addressing this gap may help clarify the biological significance of circulating IL-6, CRP, SAA, MDA, and TAC in relation to clinically assessed periodontal status.

Therefore, this study aimed to evaluate the associations between naturally occurring canine periodontal disease, clinical periodontal indices, and systemic inflammatory and oxidative stress biomarkers, specifically IL-6, CRP, SAA, MDA, and TAC.

We hypothesized that greater periodontal involvement would be associated with increased systemic inflammatory activity and oxidative imbalance, and that higher PI and GI scores would be significantly associated with circulating IL-6, CRP, SAA, MDA, and TAC levels.

## 2 Methods & materials

### 2.1 Study population and experimental design

This cross-sectional observational study was conducted on client-owned dogs presented to the Small Animal Hospital, Faculty of Veterinary Medicine, Shiraz University. A total of 131 dogs of different breeds, sexes, and age groups were enrolled following clinical and oral examinations.

Dogs were eligible for inclusion if they were routinely maintained on mixed commercial diets, including dry and wet food, and had no history of receiving mineral, antioxidant, or other nutritional supplements that could influence systemic biochemical profiles. Dogs with known or clinically suspected systemic disorders that could influence inflammatory responses or oxidative status, including chronic infectious, metabolic, endocrine, renal, hepatic, neoplastic, or inflammatory conditions, were excluded based on owner-reported medical history and clinical examination findings.

Following periodontal evaluation, dogs were assigned to either a clinically healthy group (n = 70) or a periodontal disease group (n = 61). Demographic and biological characteristics, including age, sex, breed, body weight, and skull type, were recorded for each animal. These variables were subsequently considered as potential confounding factors during statistical analyses.

Serum samples collected from all enrolled dogs were analyzed for systemic inflammatory biomarkers, including IL-6, CRP, and SAA, as well as oxidative stress-related parameters, including TAC and MDA.

Prior to participation, written informed consent was obtained from all dog owners. The experimental protocol was reviewed and approved by the Ethics Committee of Shiraz University (Code No. 4GCB2M369708), and all procedures were performed in accordance with institutional guidelines for animal welfare and research involving client-owned animals.

A schematic overview of the study workflow, including animal enrollment, periodontal assessment, serum collection, biomarker measurement, and statistical evaluation, is presented in Fig. 1.

**Fig. 1:**
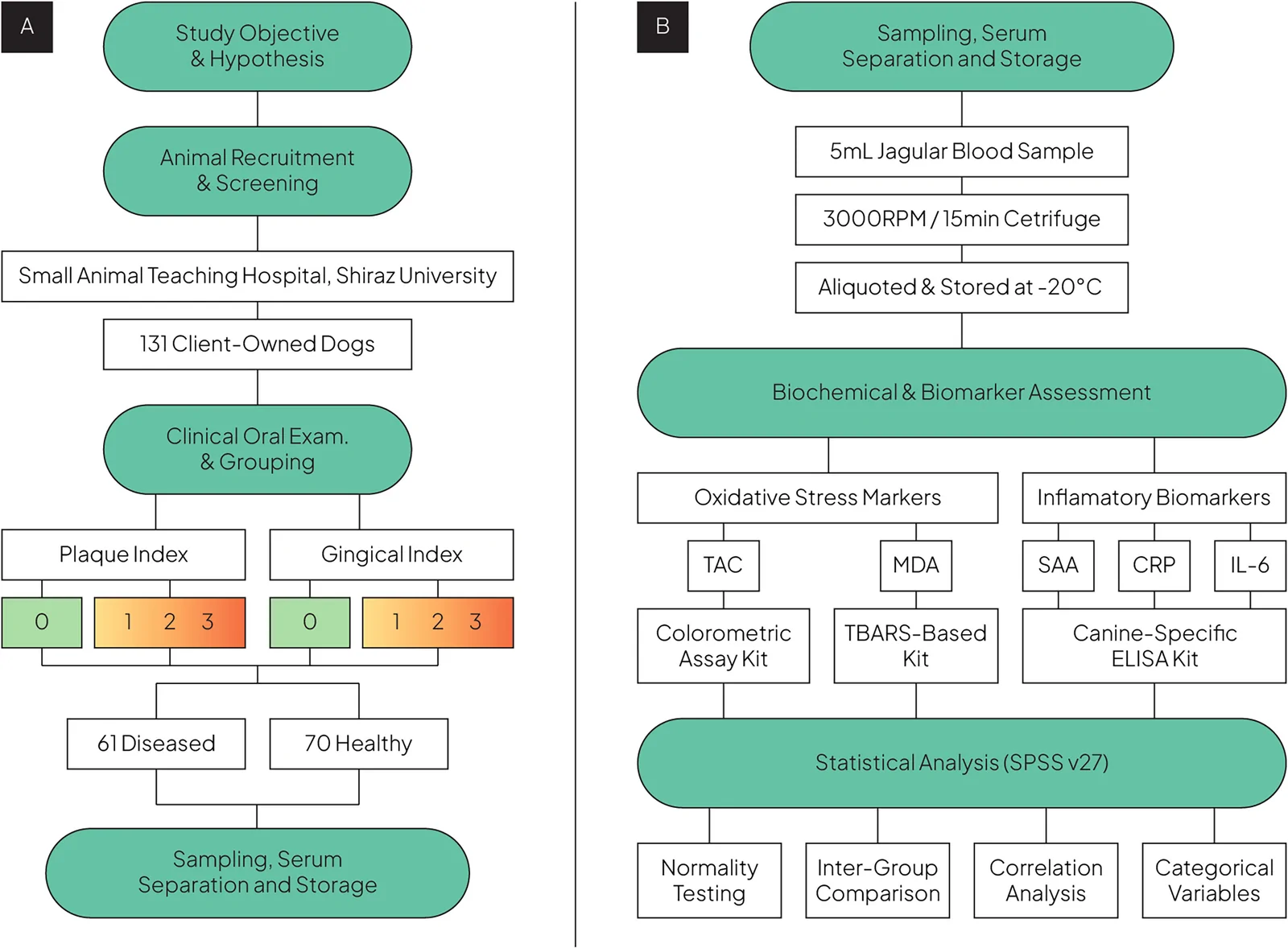
Schematic representation of the study design, including animal recruitment (client-owned dogs), classification according to periodontal status, serum collection, and biomarker analysis workflow. The diagram outlines measurement of inflammatory markers (IL-6, CRP, and SAA) and oxidative stress-related parameters (TAC and MDA), followed by statistical analysis.

### 2.2 Periodontal assessment

Oral examinations were performed in all enrolled dogs while they were awake using gentle manual restraint, without sedation or general anesthesia. Because invasive periodontal probing was not performed in privately owned animals to minimize stress and avoid unnecessary procedures, periodontal status was assessed based on visual evaluation of supragingival plaque accumulation and gingival inflammatory changes. Periodontal pocket depth, clinical attachment loss, tooth mobility, and intraoral radiographic changes were not evaluated due to the observational design and ethical considerations associated with performing invasive procedures in privately owned animals.

Clinical periodontal evaluation was performed using the PI and GI according to previously described criteria (Löe, 1967). Both indices were recorded on a four-point ordinal scale ranging from 0 to 3, with higher scores indicating greater plaque accumulation or gingival inflammatory severity. The criteria used for PI and GI scoring are presented in Supplementary Table S1.

Dogs were classified into clinically healthy and periodontal disease groups based on their clinical periodontal findings. Animals showing no visible plaque accumulation and no signs of gingival inflammation (PI = 0 and GI = 0) were considered clinically healthy. Dogs exhibiting plaque accumulation (PI ≥ 1), gingival inflammation (GI ≥ 1), or both conditions were assigned to the periodontal disease group.

Individual PI and GI scores were recorded for each dog and subsequently used for comparative analyses between groups, evaluation of periodontal severity categories, and correlation analyses with serum inflammatory and oxidative stress biomarkers.

### 2.3 Blood collection and serum preparation

Blood samples were obtained from all dogs by jugular venipuncture between 8:00 a.m. and 12:00 p.m. Approximately 5 mL of whole blood was collected into serum-separator tubes and allowed to clot at room temperature. Samples were subsequently centrifuged at 3000 rpm for 15 min to obtain serum.

The separated serum was transferred into sterile microcentrifuge tubes and stored at −20 °C until biochemical analyses were performed.

### 2.4 Measurement of inflammatory biomarkers

Serum IL-6 concentrations were determined using a canine-specific sandwich enzyme-linked immunosorbent assay (ELISA) kit (Cusabio, Shanghai, China; Catalog No. CSB-E11260c). The assay sensitivity was 0.039 ng/mL, with a detection range of 0.156–1.00 ng/mL. According to the manufacturer, intra-assay and inter-assay coefficients of variation were below 8% and 10%, respectively.

Serum CRP concentrations were measured using a commercial canine ELISA kit (Shanghai Crystal Day Biotech Company, China; Catalog No. CRP E0124ca). The analytical sensitivity of the assay was 7.8 pg/mL, and intra- and inter-assay coefficients of variation were below 8% and 10%, respectively.

Serum amyloid A concentrations were quantified using a canine-specific ELISA kit (Shanghai Crystal Day Biotech Company, China; Catalog No. SAA E0125ca). The assay sensitivity was 0.156 mg/mL, with intra-assay and inter-assay coefficients of variation below 8% and 10%, respectively.

### 2.5 Assessment of oxidative stress biomarkers

Serum TAC was measured using a commercial colorimetric assay kit (ZellBio GmbH, Germany). The assay is based on the reduction of a chromogenic substrate, and absorbance was measured at 450 nm using an ELISA microplate reader. The analytical sensitivity of the assay was 0.1 mmol/L. Reported intra-assay and inter-assay coefficients of variation were 3.4% and 4.2%, respectively.

Serum MDA, a marker of lipid peroxidation, was determined using a thiobarbituric acid reactive substances (TBARS)-based commercial kit (ZellBio GmbH, Germany). In this method, MDA reacts with thiobarbituric acid under acidic and high-temperature conditions to form a colored complex. Absorbance was measured at 535 nm. The assay sensitivity was 0.1 μmol/L, and the inter-assay coefficient of variation was reported as 5.8%.

### 2.6 Statistical analysis

Statistical analyses were performed using SPSS software version 27.0 (IBM Corp., Armonk, NY, USA). Data distribution was evaluated using the Shapiro–Wilk test. Because several variables deviated from normal distribution, non-parametric statistical approaches were applied.

Differences in demographic variables, periodontal indices, and serum biomarker concentrations between healthy and periodontal disease groups were assessed using the Mann–Whitney U test. The Kruskal–Wallis test was used to compare biomarker concentrations among PI and GI severity categories, followed by Dunn’s post-hoc analysis with Bonferroni correction when significant differences were detected.

Associations between periodontal indices and serum biomarkers were examined using Spearman’s rank correlation analysis. Categorical variables were evaluated using chi-square tests where appropriate. Continuous variables were expressed as median and interquartile range (IQR), while categorical data were presented as frequency and percentage.

To determine whether periodontal disease status was independently associated with serum biomarkers after accounting for potential confounders, multivariable generalized linear models were constructed using a Gamma distribution with a log-link function. Disease status was entered as the primary explanatory variable, while age, sex, body weight, and skull type were included as covariates.

A sensitivity power analysis was conducted using SPSS Power Analysis based on the final available sample size (70 healthy dogs and 61 dogs with periodontal disease). Assuming a two-sided independent-samples comparison, α = 0.05, standardized effect size of 0.50, and equal variance assumptions, the estimated achieved power was 80.9%. Because recruitment depended on the availability of eligible client-owned animals during the study period, this analysis was considered a sensitivity analysis rather than an a priori sample size calculation.

A two-tailed P value <0.05 was considered statistically significant.

## 3 Results

### 3.1 Characteristics of the study population

The demographic and clinical characteristics of enrolled dogs are summarized in Table 1. The periodontal disease group was significantly older than the healthy group (P = 0.023) and showed lower median body weight (P = 0.001). Sex distribution did not differ between groups (P = 0.862). However, differences were observed in body size distribution, with a higher proportion of small-sized dogs in the periodontal disease group (P < 0.001). Skull type distribution showed a borderline difference between groups (P = 0.055).

**Table 1:** Demographic characteristics and potential confounders associated with periodontal status in enrolled dogs.

**Characteristic**	**p**	**Periodontal disease dogs (n=61)**	**Statistical test**	**P value**
Age (months), median (IQR)	36 (48)	48 (54)	Mann–Whitney U	0.023
Body weight (kg), median (IQR)	22.06 (2.60)	20.13 (15.60)	Mann–Whitney U	0.001
Male, n (%)	32 (45.7)	26 (42.6)	χ² test	0.862
Female, n (%)	38 (54.3)	29 (47.5)		
Not recorded sex, n (%)	0 (0)	6 (9.8)		
Small size, n (%)	5 (7.1)	24 (39.3)	χ² test	<0.001
Medium size, n (%)	65 (92.9)	31 (50.8)		
Large size, n (%)	0 (0.0)	4 (6.6)		
Giant size, n (%)	0 (0.0)	2 (3.3)		
Mesocephalic skull, n (%)	65 (92.9)	49 (80.3)	χ² test	0.055
Brachycephalic skull, n (%)	5 (7.1)	9 (14.8)		
Dolichocephalic skull, n (%)	0 (0.0)	3 (4.9)		

The chi-square test for sex distribution was performed using dogs with recorded sex; six dogs in the periodontal disease group had no sex information recorded. Small size (< 10 kg), medium size (10- < 25 kg), large size (25- < 40 kg), or giant size (≥40 kg) (Fahie et al., 2018).

### 3.2 Comparison of periodontal indices and serum biomarkers between healthy and periodontal disease dogs

Dogs affected by periodontal disease exhibited a consistent pattern of elevated clinical and biochemical markers compared with healthy controls. Specifically, PI and GI scores were significantly increased in diseased animals, accompanied by marked elevations in serum IL-6, CRP, SAA, and MDA concentrations, while TAC was significantly reduced (Table 2).

**Table 2:** Comparison of periodontal indices and serum biomarkers between healthy and periodontal disease dogs.

**Variable**	**IQR Median (25–75%)**		**Mann-Whitney U**	**Z Value**	**P Value**	**Effect r**
	**Healthy**	**Diseased**				
IL-6 (ng/mL)	0.29 (0.26–0.33)	0.75 (0.53–0.85)	0.00	−9.86	<0.001	0.861
CRP (µg/mL)	3.17 (2.91–3.50)	7.49 (5.95–12.15)	0.00	−9.85	<0.001	0.861
TAC (µmol/L)	3.32 (2.36–3.82)	3.01 (2.01–3.33)	1415.00	−3.32	0.001	0.290
MDA (µmol/L)	0.55 (0.52–0.57)	0.59 (0.56–0.61)	904.00	−5.71	<0.001	0.499
SAA (mg/mL)	6.58 (6.37–6.81)	14.52 (11.81–17.90)	0.00	−9.85	<0.001	0.861

Interleukin-6 (IL-6); C-reactive protein (CRP); Serum amyloid A (SAA); Malondialdehyde (MDA); Total antioxidant capacity (TAC). Statistical significance was set at p < 0.05.

The largest effect sizes were observed for IL-6, CRP, and SAA (r = 0.86), indicating substantial differences between dogs with and without periodontal disease status. MDA shows a moderate-to-large difference (r = 0.50), whereas TAC shows a smaller difference (r = 0.29). These effect sizes reinforce that the inflammatory-marker findings are much stronger than the TAC signal.

### 3.3 Serum biomarker alterations according to plaque index severity

The Kruskal–Wallis test revealed significant differences in serum biomarker concentrations across plaque index severity categories. Significant variations were observed in IL-6, CRP, SAA, MDA (all p < 0.001), and TAC (p = 0.010), indicating that periodontal severity based on plaque accumulation is associated with systemic inflammatory and oxidative stress alterations.

Overall, increasing plaque index severity was associated with a progressive increase in IL-6, CRP, SAA, and MDA levels, while TAC showed a decreasing trend across severity categories. These findings suggest a severity-related association between plaque accumulation and systemic inflammatory burden as well as oxidative imbalance.

### 3.4 Serum biomarker alterations according to gingival index severity

The Kruskal–Wallis test demonstrated significant differences in serum biomarker concentrations across gingival index severity categories. Significant variations were observed in IL-6, CRP, SAA, MDA (all p < 0.001), and TAC (p = 0.033), indicating that gingival inflammation severity is associated with systemic inflammatory and oxidative stress alterations.

Overall, increasing gingival index severity was associated with elevated serum levels of IL-6, CRP, SAA, and MDA, whereas TAC showed a decreasing trend across severity categories. These findings suggest a positive association between gingival inflammation severity and systemic inflammatory burden, along with oxidative imbalance, although the pattern for TAC was less consistent compared with inflammatory biomarkers.

### 3.5 Correlation between periodontal indices and serum biomarkers

Spearman correlation analysis revealed significant associations between periodontal indices and serum biomarkers (all p < 0.05), with variation in effect sizes across parameters.

For plaque index, the strongest positive correlations were observed with CRP (r = 0.893), SAA (r = 0.893), and IL-6 (r = 0.883). Plaque index also showed a moderate positive correlation with MDA (r = 0.449), whereas TAC exhibited a weak negative correlation (r = −0.266).

Similarly, gingival index showed positive correlations with IL-6 (r = 0.687), CRP (r = 0.678), SAA (r = 0.680), and MDA (r = 0.343), along with a weak negative correlation with TAC (r = −0.222). Overall, correlation coefficients for gingival index were lower than those observed for plaque index across most biomarkers.

A heatmap representation of these associations is presented in Fig. 2, illustrating strong positive correlations between periodontal indices and inflammatory markers (IL-6, CRP, SAA), moderate correlations with MDA, and weak negative correlations with TAC.

**Fig. 2:**
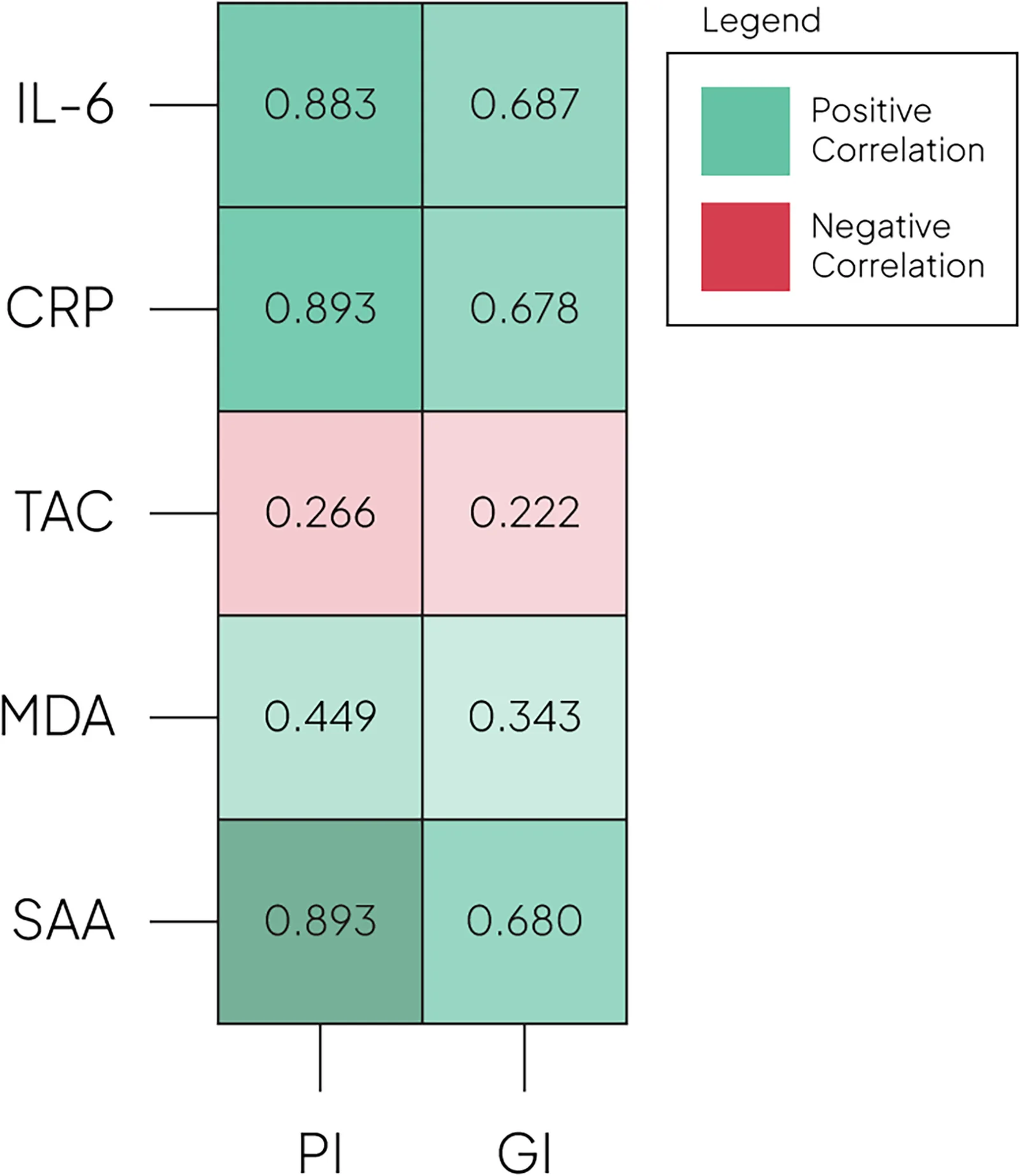
Heatmap of Spearman correlation coefficients between periodontal indices (plaque index [PI] and gingival index [GI]) and serum inflammatory and oxidative stress biomarkers in dogs, including interleukin-6 (IL-6), c-reactive protein (CRP), serum amyloid A (SAA), malondialdehyde (MDA), total antioxidant capacity (TAC). Positive correlations are shown in green, whereas negative correlations are shown in red. Each cell represents the corresponding correlation coefficient (r value), with color intensity reflecting the strength of the association.

### 3.6 Relationship between plaque index and gingival index

A significant positive correlation was detected between plaque index and gingival index (r = 0.606, p < 0.001), indicating that higher plaque accumulation is associated with increased gingival inflammation severity.

Fig. 3 illustrates the relationship between PI and GI in dogs with periodontal disease. Fig. 3 illustrates the relationship between PI and GI in dogs with periodontal disease. Each cell shows the number of dogs within the corresponding PI and GI category, indicating a moderate positive association between plaque accumulation and gingival inflammation.

**Fig. 3:**
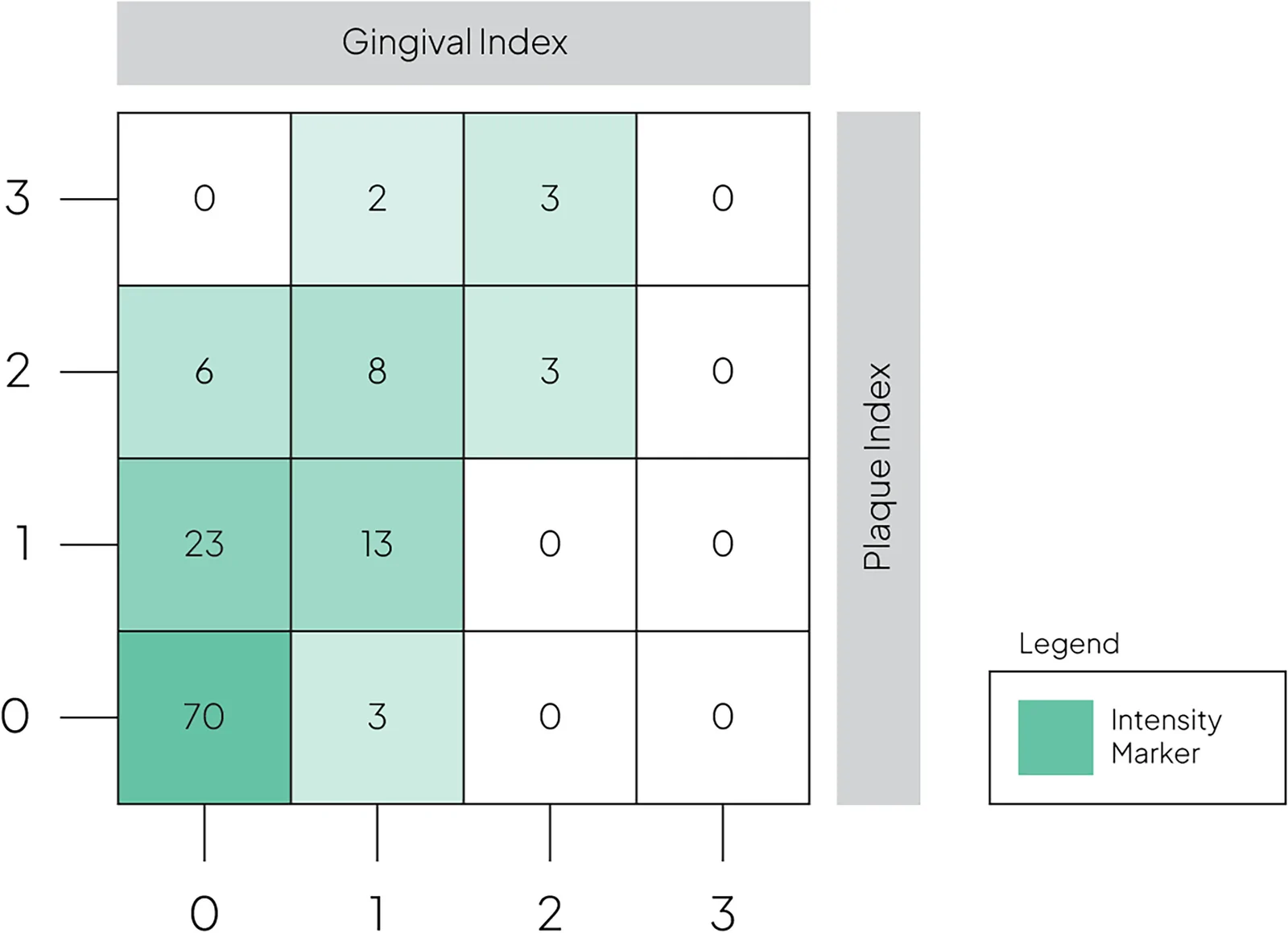
Contingency-table heatmap showing correlation between plaque index (PI) and gingival index (GI) in dogs with periodontal disease. Each cell represents the number of individual dogs within the corresponding PI and GI category. A significant positive correlation was observed (Spearman’s r = 0.606, p < 0.001), indicating that higher plaque accumulation is associated with increased gingival inflammation severity.

### 3.7 Association between age and periodontal status

Age showed weak but statistically significant positive correlations with both plaque index (r = 0.266, p = 0.002) and gingival index (r = 0.189, p = 0.032).

As illustrated in Fig. 4, mean periodontal indices increased across age categories. The increase in plaque index was more pronounced compared with gingival index, indicating a stronger age-related trend for plaque accumulation.

**Fig. 4:**
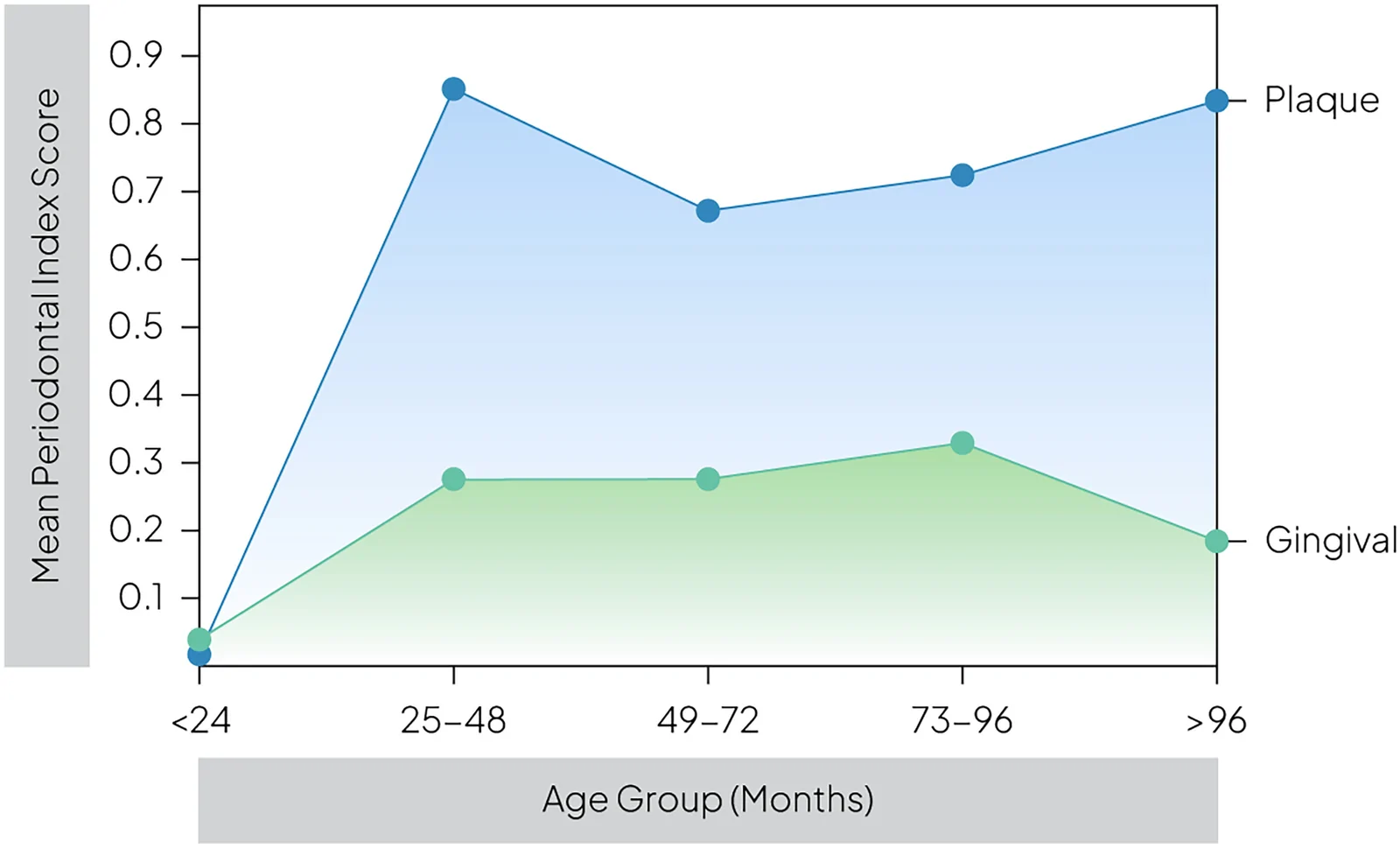
Age-related variation in periodontal indices in dogs. Mean plaque index (PI) and gingival index (GI) across age groups (<24, 25–48, 49–72, 73–96, and >96 months). Data are presented as mean values for each age category. The figure shows an increasing trend in periodontal indices with age, with a more pronounced pattern for plaque index compared with gingival index.

### 3.8 Association of sex with periodontal indices and serum biomarkers

No significant differences were observed between male and female dogs in serum concentrations of IL-6, CRP, SAA, MDA, or TAC (all p > 0.05). Effect size analysis further confirmed the absence of meaningful sex-related differences in systemic inflammatory and oxidative stress biomarkers, with all confidence intervals crossing the null value (Fig. 5).

**Fig. 5:**
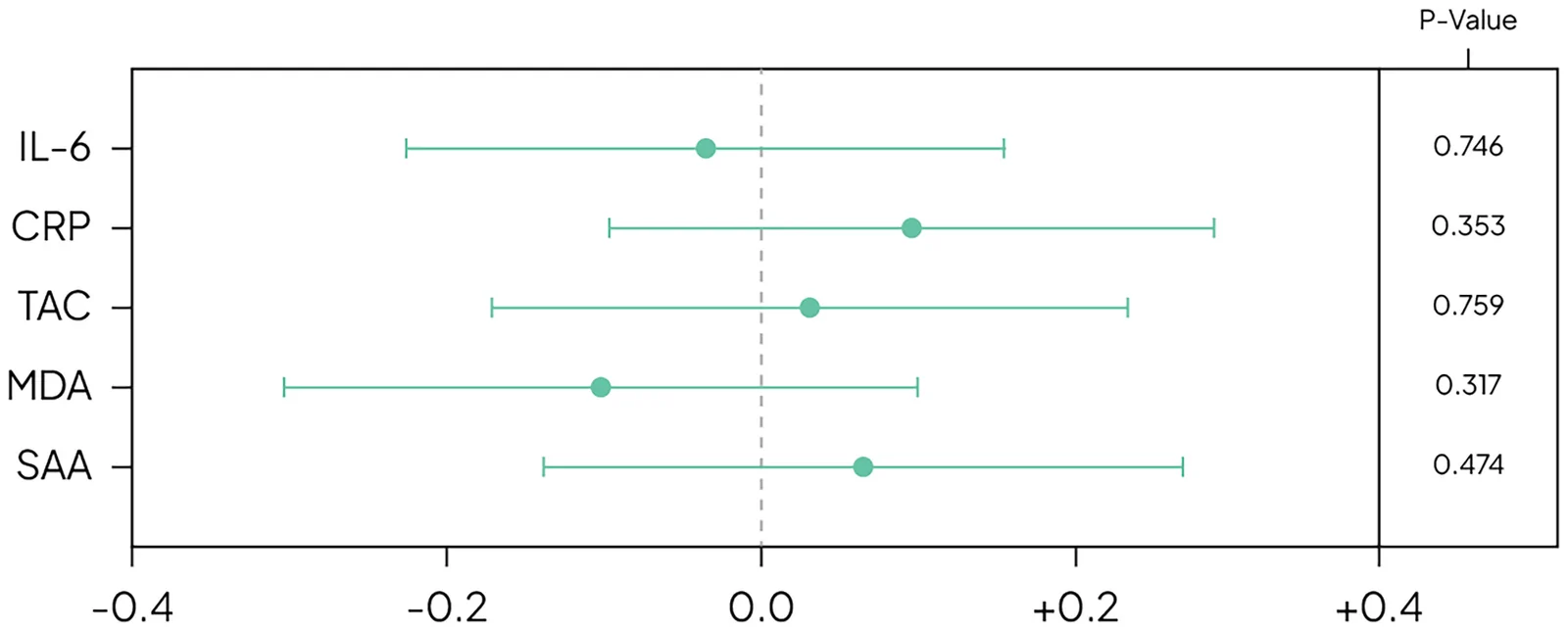
Forest plot illustrating sex-related differences in serum inflammatory and oxidative stress biomarkers in dogs. Effect size estimates (rank-biserial correlation coefficients) with 95% confidence intervals are shown for interleukin-6 (IL-6), c-reactive protein (CRP), serum amyloid A (SAA), malondialdehyde (MDA) and total antioxidant capacity (TAC). Positive values indicate higher levels in female dogs, whereas negative values indicate higher levels in male dogs. All confidence intervals crossed the null line, indicating no statistically significant sex-related differences for any biomarker (Mann–Whitney U test, all p > 0.05).

Similarly, sex was not significantly associated with periodontal indices. Chi-square analysis revealed no significant relationship between sex and gingival index (Pearson χ² = 3.093, df = 2, p = 0.213) or plaque index (Pearson χ² = 1.406, df = 3, p = 0.704). Likelihood ratio and linear-by-linear association analyses confirmed the absence of significant sex-related differences in periodontal status distribution (Fig. 6).

**Fig. 6:**
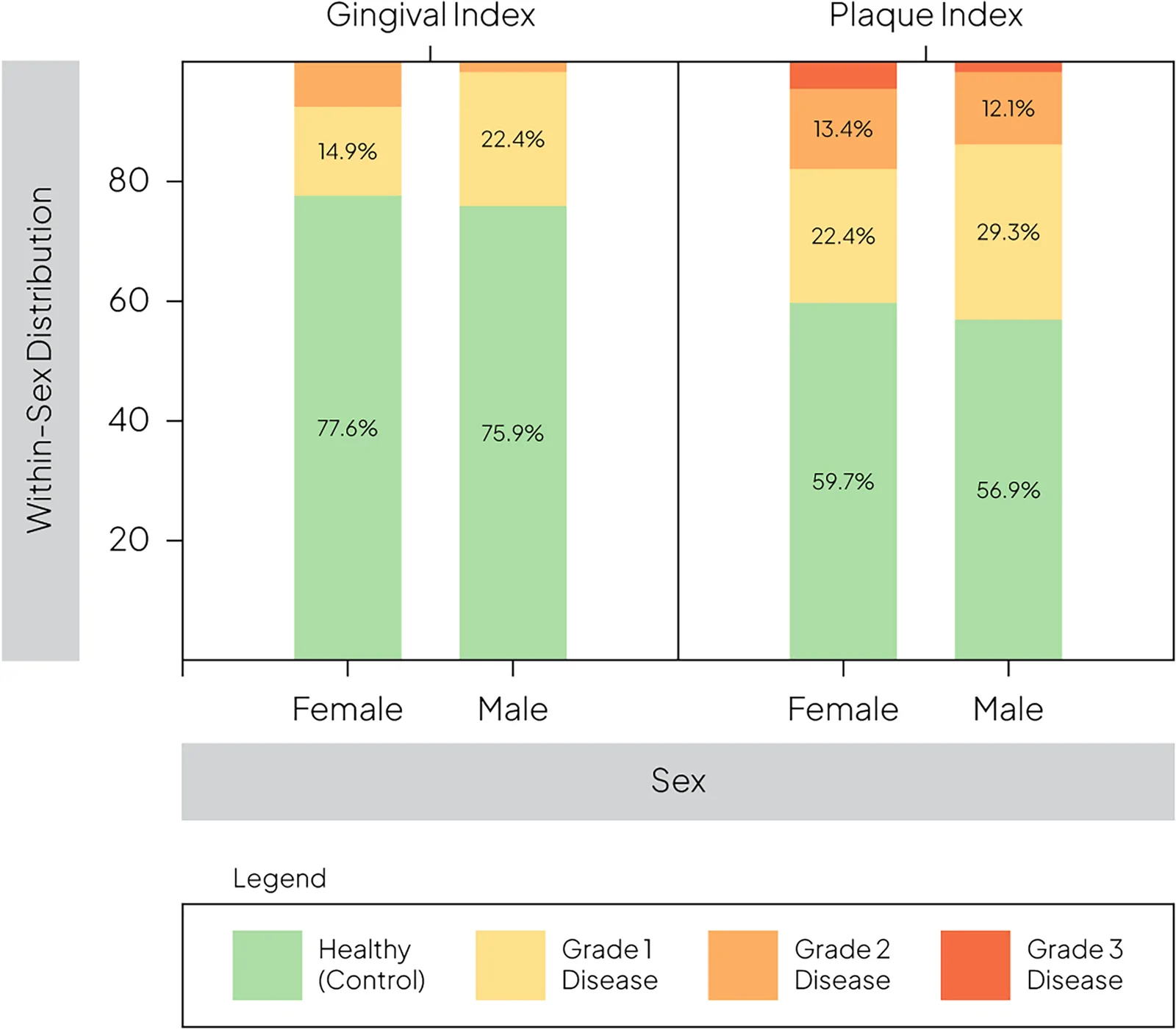
Stacked bar charts showing sex-related distribution of periodontal status based on gingival index and plaque index categories (healthy/control and Grades 1–3). The distribution of periodontal categories within each sex is presented for both indices. No statistically significant differences were observed between males and females for either gingival or plaque index, as confirmed by Pearson chi-square, likelihood ratio, and linear-by-linear association tests (all p > 0.05).

### 3.9 Multivariable adjustment for potential confounding

To evaluate whether the association between periodontal disease status and serum inflammatory and oxidative stress biomarkers was independent of potential demographic confounders, multivariable generalized linear models with gamma distribution and log-link function were performed. Disease status was included as the primary predictor, while age, sex, body weight, and skull type were entered as covariates.

After adjustment, periodontal disease status remained significantly associated with all evaluated biomarkers included in the models. Diseased dogs exhibited higher serum concentrations of IL-6 (Exp(B)=2.430, 95% CI: 2.246–2.629, P < 0.001), CRP (Exp(B)=2.847, 95% CI: 2.541–3.189, P < 0.001), SAA (Exp(B)=2.427, 95% CI: 2.231–2.641, P < 0.001), and MDA (Exp(B)=1.059, 95% CI: 1.040–1.079, P < 0.001) compared with healthy dogs. These findings corresponded to adjusted increases of approximately 143%, 185%, 143%, and 6% in IL-6, CRP, SAA, and MDA concentrations, respectively, in dogs with periodontal disease. Conversely, TAC showed a modest but significant inverse association with periodontal disease status (Exp(B)=0.885, 95% CI: 0.797–0.983, P = 0.023), indicating an approximately 11.5% reduction in adjusted TAC levels among affected dogs (Table 3).

**Table 3:** Multivariable generalized linear models evaluating the association between periodontal disease status and serum biomarkers after adjustment for demographic confounders.

**Biomarker**	**Adjusted Exp(B)**	**95% CI**	**Wald χ²**	**P-value**	**Adjusted difference (%)**
IL-6	2.430	2.246–2.629	488.535	<0.001	+143.0
CRP	2.847	2.541–3.189	325.631	<0.001	+184.7
TAC	0.885	0.797–0.983	5.173	0.023	−11.5
MDA	1.059	1.040–1.079	36.083	<0.001	+5.9
SAA	2.427	2.231–2.641	423.627	<0.001	+142.7

Interleukin-6 (IL-6); C-reactive protein (CRP); Serum amyloid A (SAA); Malondialdehyde (MDA); Total antioxidant capacity (TAC). Statistical significance was set at p < 0.05.

Among the covariates, age was independently associated with serum IL-6 (P = 0.009) and CRP (P = 0.019), while its association with TAC showed borderline significance (P = 0.048). No significant associations were observed between sex, skull type, or body weight and the evaluated biomarkers. Detailed results of covariate effects are presented in Supplementary Table S2.

## 4 Discussion

The present study demonstrates that canine periodontal disease status is associated with significant systemic inflammatory and oxidative stress-related alterations, as evidenced by increased serum concentrations of IL-6, CRP, SAA, and MDA, together with reduced TAC levels. Rather than establishing a causal relationship between oral disease and systemic alterations, these findings indicate that clinically detectable periodontal changes are accompanied by measurable systemic biochemical responses. This interpretation is particularly relevant because the evaluated biomarkers represent general inflammatory and oxidative stress pathways and are not exclusively specific to periodontal tissues. Thus, the present findings should be interpreted as evidence of concurrent systemic biochemical alterations associated with clinically detectable periodontal disease rather than as evidence of a direct systemic consequence of periodontal disease.

Previous studies have suggested that periodontal inflammation may be accompanied by systemic immune activation through increased exposure to microbial products, inflammatory mediators, and oxidative challenges. However, because the present investigation employed a cross-sectional design, the observed associations should be interpreted as concurrent alterations rather than evidence that periodontal disease directly initiates systemic inflammation. The current findings support the concept that periodontal pathology in dogs may represent one component of a broader inflammatory–oxidative network involving local oral changes and systemic biological responses (Foroughi et al., 2025).

Importantly, both clinical periodontal indices (plaque index and gingival index) showed consistent associations with systemic biomarkers, supporting a close relationship between clinical periodontal status and systemic inflammatory activity. Strong positive correlations were observed between plaque index and IL-6, CRP, and SAA, whereas gingival index also demonstrated significant positive associations with these biomarkers. The stronger correlations observed for plaque accumulation compared with gingival inflammation suggest that the amount of microbial plaque burden may be more closely related to systemic inflammatory marker variation in this population. However, this finding should not be interpreted as evidence of a direct causal effect of plaque accumulation on circulating biomarkers, because other biological and environmental factors may contribute to these associations.

The strong relationships between inflammatory biomarkers and periodontal indices, together with the relatively weaker associations observed for oxidative markers, indicate that systemic inflammatory activation may represent a more consistent feature of clinically detectable periodontal alterations than oxidative imbalance. Similar observations have been reported in human and veterinary studies, where inflammatory mediators frequently demonstrate stronger associations with periodontal severity compared with oxidative stress-related parameters (Angiolani et al., 2025; Baltacıoğlu et al., 2014; Song et al., 2019).

IL-6 is a central cytokine involved in the regulation of inflammatory and immune responses and plays a key role in stimulating acute-phase protein synthesis (Zhang et al., 2026). In periodontal disease, sustained exposure to bacterial components and dysbiotic microbial communities may trigger immune activation and cytokine release from both infiltrating immune cells and resident gingival cells (Sedghi et al., 2021). In the present study, IL-6 concentrations were markedly higher in dogs classified with periodontal disease and remained significantly associated with disease status after adjustment for age, sex, body weight, and skull type. This finding suggests that the relationship between periodontal status and circulating IL-6 was not solely explained by demographic differences between groups.

Previous experimental and clinical investigations have similarly highlighted IL-6 as a relevant marker of inflammatory activity associated with periodontal conditions (Khan et al., 2023; Relvas et al., 2024). Nevertheless, because IL-6 is involved in numerous inflammatory and age-related conditions, its elevation should be interpreted as an indicator of systemic inflammatory activation rather than a periodontal-specific biomarker.

CRP, a major acute-phase protein synthesized primarily by hepatocytes in response to inflammatory cytokines, is widely used as an indicator of systemic inflammatory activity (Zhou et al., 2024). In this study, CRP concentrations were significantly elevated in dogs with periodontal disease and showed one of the strongest associations with periodontal status among the evaluated biomarkers. The persistence of this association after multivariable adjustment further supports the relationship between clinical periodontal alterations and systemic inflammatory profile.

Previous studies have reported increased CRP concentrations in association with periodontal inflammation, although variability among studies may result from differences in disease definitions, population characteristics, age distribution, and methodological approaches (Moradi Haghgoo et al., 2023; Rodean et al., 2025; Valiyaveetil Karattil et al., 2022). In dogs, interpretation of CRP should also consider that it represents a nonspecific acute-phase response and may reflect inflammatory processes occurring outside the oral cavity.

Serum amyloid A is another highly sensitive acute-phase protein primarily regulated by inflammatory cytokines, including IL-6 (Papareddy & Herwald, 2025). In the present study, SAA concentrations were significantly increased in dogs with periodontal disease and demonstrated strong associations with both plaque accumulation and gingival inflammation. The magnitude of SAA alteration suggests that this biomarker may be a sensitive indicator of systemic inflammatory status in dogs exhibiting clinical periodontal changes; however, its diagnostic specificity for oral disease remains uncertain.

Given the limited information available regarding SAA responses in canine periodontal conditions, further longitudinal studies evaluating biomarker changes before and after periodontal treatment are required to determine whether alterations in SAA reflect reversible oral inflammation-associated responses or broader systemic inflammatory processes. Previous veterinary studies have demonstrated the value of SAA as an acute-phase marker in inflammatory disorders, supporting its potential relevance in periodontal research (Christensen et al., 2013; Oikonomidis et al., 2025).

Oxidative stress is considered an important component of periodontal inflammatory responses, reflecting an imbalance between reactive oxygen species generation and antioxidant defense mechanisms (Patil et al., 2024). In the present study, dogs with periodontal disease exhibited increased serum MDA concentrations and reduced TAC levels, indicating the presence of systemic oxidative alterations associated with clinical periodontal status. However, these findings should be interpreted cautiously because oxidative stress biomarkers are influenced by multiple physiological and pathological conditions and are not specific indicators of oral inflammation.

MDA, a commonly used marker of lipid peroxidation, reflects oxidative damage resulting from excessive reactive oxygen species production. In the current study, serum MDA concentrations were significantly higher in dogs with periodontal disease compared with healthy animals and remained independently associated with periodontal status after adjustment for demographic variables. However, the magnitude of the MDA alteration was considerably smaller than that observed for inflammatory biomarkers, and the relationship between MDA and periodontal indices was moderate compared with the strong associations observed for IL-6, CRP, and SAA. This difference suggests that oxidative damage may represent a secondary or variable component of the systemic response associated with periodontal alterations rather than the predominant biological signal.

Previous studies have reported increased oxidative stress markers in periodontal disorders, supporting the involvement of reactive oxygen species in periodontal inflammatory pathways (Andreu et al., 2021). Nevertheless, the relatively modest variation in MDA across severity categories in the present study may indicate that lipid peroxidation does not increase proportionally with visible periodontal severity. Alternatively, this pattern may reflect biological heterogeneity among client-owned dogs, including differences in age, breed-related susceptibility, metabolic status, and environmental factors.

TAC represents the overall antioxidant capacity of biological fluids and provides an integrated measure of endogenous antioxidant defense (Mohideen et al., 2023). In this study, TAC concentrations were significantly lower in dogs with periodontal disease compared with healthy controls, and multivariable analysis demonstrated an inverse association between periodontal disease status and TAC levels. The reduction in TAC may reflect increased utilization of antioxidant systems in response to chronic inflammatory stimulation and oxidative challenges associated with periodontal alterations (Moosavian et al., 2024; Pavlica et al., 2004). However, the relatively small effect size of TAC compared with inflammatory biomarkers suggests that antioxidant depletion may be more variable among individuals.

Interestingly, TAC differences were not consistently maintained across all periodontal severity categories. This finding should not be interpreted as evidence of restoration of antioxidant balance in advanced disease, because some severity subgroups contained a limited number of animals, reducing statistical power. In addition, biological adaptation mechanisms and individual variation in antioxidant capacity may influence circulating TAC concentrations.

The combined evaluation of inflammatory and oxidative biomarkers provides a more comprehensive understanding of the systemic biological profile associated with canine periodontal status. The current results indicate that inflammatory markers (IL-6, CRP, and SAA) demonstrated the strongest and most consistent associations with periodontal indices, whereas oxidative stress-related parameters (MDA and TAC) showed comparatively weaker relationships. This pattern suggests that systemic inflammatory activation may be a more reliable measurable feature of clinical periodontal alterations, while oxidative imbalance may represent a complementary but less specific pathway (Chen et al., 2019). Nevertheless, the present biomarker panel represents only selected components of the systemic response, and additional inflammatory, oxidative, metabolic, or tissue-specific biomarkers may provide a broader characterization of periodontal–systemic interactions.

The observed associations between plaque accumulation, gingival inflammation, and circulating biomarkers support the concept that local oral inflammatory changes may coexist with systemic inflammatory and oxidative alterations. However, because the evaluated biomarkers are involved in numerous systemic conditions, these findings should not be interpreted as evidence that periodontal disease is the sole source of systemic inflammation. Instead, they indicate that dogs with clinically detectable periodontal alterations exhibit a distinct systemic biochemical profile that warrants further investigation.

Age showed a weak but statistically significant association with periodontal indices, suggesting that aging may contribute to increased periodontal burden in dogs, consistent with previous observations in veterinary and human populations (Tadjoedin et al., 2017; Uçan Yarkaç et al., 2024). The periodontal disease group in the present study was also older than the healthy group, highlighting age as an important potential confounder. Importantly, multivariable modeling demonstrated that periodontal status remained significantly associated with all evaluated biomarkers after adjustment for age and other demographic variables, although age independently influenced IL-6 and CRP concentrations.

These findings emphasize the importance of considering age when interpreting systemic inflammatory biomarkers in canine periodontal research, as aging itself may contribute to chronic low-grade inflammatory activation. Nevertheless, the persistence of periodontal associations after statistical adjustment suggests that the observed biomarker differences cannot be fully explained by age alone.

No significant differences were detected between male and female dogs regarding periodontal indices or serum biomarker concentrations. Similarly, sex was not independently associated with biomarker concentrations in adjusted analyses, suggesting that sex-related variation did not substantially influence the systemic inflammatory and oxidative profiles observed in this study. This finding is consistent with previous veterinary studies reporting limited influence of sex on periodontal-associated inflammatory alterations (Carreira et al., 2015).

Body size and skull type were also considered as potential confounding factors because breed-associated anatomical characteristics, particularly brachycephalic conformation and smaller body size, may influence periodontal susceptibility due to dental crowding and altered oral anatomy. Although significant differences in body size distribution were observed between groups, adjustment for body weight and skull type did not eliminate the association between periodontal status and serum biomarkers. These results suggest that anatomical and demographic differences may contribute to periodontal susceptibility but are unlikely to fully account for the systemic biomarker alterations detected in the present study.

The present study has several methodological strengths. First, the inclusion of demographic variables and application of multivariable generalized linear models allowed evaluation of periodontal–biomarker associations while accounting for important potential confounders. Second, the combined assessment of inflammatory and oxidative stress markers provided a broader characterization of systemic biochemical changes associated with clinical periodontal status. Third, the use of standardized PI and GI scoring enabled evaluation of periodontal severity beyond simple case–control comparisons.

Several limitations should be acknowledged. The cross-sectional design limits causal interpretation, and the observed associations do not establish whether periodontal disease drives systemic alterations, systemic conditions contribute to oral changes, or both are influenced by shared factors. Periodontal assessment was based on visual PI and GI scoring without periodontal probing, attachment loss evaluation, tooth mobility assessment, or intraoral radiography; therefore, it reflects clinical plaque accumulation and gingival inflammation rather than complete periodontal tissue destruction. Although dogs with known systemic disorders were excluded based on medical history and clinical examination, comprehensive screening for subclinical conditions was not performed, and the evaluated biomarkers are not specific to oral inflammation. Finally, the use of client-owned dogs with naturally occurring disease introduces biological variability related to breed, lifestyle, and previous oral care. Although age, sex, body weight, and skull type were accounted for in the multivariable analyses, the heterogeneity of the study population and the absence of systematic information on some potentially relevant factors mean that residual confounding cannot be completely excluded. The limited number of animals in some advanced severity categories may also have reduced statistical power for subgroup analyses, particularly for oxidative stress markers.

Future studies should employ longitudinal designs to evaluate biomarker changes before and after periodontal treatment, including dental cleaning and periodontal therapy, to determine whether systemic inflammatory and oxidative alterations are reversible following improvement of oral health. Integration of serum biomarkers with local oral samples, including gingival crevicular fluid or saliva, together with molecular inflammatory profiling, may further clarify the relationship between local periodontal inflammation and systemic biological responses.

## 5 Conclusion

In conclusion, the present findings indicate that clinically detectable periodontal alterations in dogs are associated with measurable systemic inflammatory and oxidative stress-related changes. While inflammatory biomarkers such as IL-6, CRP, and SAA demonstrated the strongest associations with periodontal status, oxidative stress markers provided additional evidence of altered systemic homeostasis. These results support the potential value of combined clinical and biochemical assessment in canine periodontal research while emphasizing the need for longitudinal studies to clarify biological mechanisms and clinical applicability.

## Declarations

**Ethical approval:** All applicable international, national, and/or institutional guidelines were followed.

## Funding

This study was supported by 10.13039/501100005071Shiraz University, Shiraz, Iran, as part of a graduate thesis (Grant number 0GRC1M369708). The authors gratefully acknowledge the financial support provided by Shiraz University.

## CRediT authorship contribution statement

**Negin Sharei:** Visualization, Software, Resources, Methodology, Investigation. **Nooshin Derakhshandeh:** Validation, Project administration, Methodology, Investigation, Conceptualization. **Milad Faraji:** Writing – original draft, Resources, Investigation, Formal analysis. **Artin Sheibani:** Validation, Formal analysis, Data curation. **Saeed Nazifi:** Writing – review & editing, Visualization, Validation, Supervision, Resources, Methodology, Funding acquisition.

## Declaration of competing interest

The authors declare that they have no competing interests.

## Data Availability

The datasets generated and/or analyzed during the current study are available from the corresponding author upon reasonable request.
