# Supplementary material for: Clinical periodontal indices are associated with systemic inflammatory and oxidative stress biomarkers in dogs with periodontal disease
Source: Vet Anim Sci. 2026 Sep 24;34:100882. doi: 10.1016/j.vas.2026.100882 (PMC13636072; doi:10.1016/j.vas.2026.100882)
Supplement: Supplementary file 1 [file mmc1.docx]

Table S 1. Clinical criteria used for assessment of plaque accumulation and gingival inflammation in dogs

| **Index** | **Score** | **Clinical criteria** |
| --- | --- | --- |
| **Plaque Index (PI)** | 0 | No visible plaque accumulation |
|  | 1 | Detectable plaque accumulation along the gingival margin |
|  | 2 | Moderate plaque accumulation extending along the gingival margin |
|  | 3 | Extensive plaque accumulation involving a large proportion of the tooth surface |
| **Gingival Index (GI)** | 0 | Clinically healthy gingiva without visible inflammatory changes |
|  | 1 | Mild gingival inflammation characterized by slight erythema and edema |
|  | 2 | Moderate inflammation with evident erythema, edema, and altered gingival appearance |
|  | 3 | Severe inflammation characterized by marked erythema, edema, ulceration, and/or spontaneous bleeding |

Table S 2. Association of demographic covariates with serum inflammatory and oxidative stress biomarkers in multivariable generalized linear models

| **Biomarker** | **Age P value** | **Weight P value** | **Sex P value** | **Skull type P value** |
| --- | --- | --- | --- | --- |
| IL-6 | 0.009 | 0.228 | 0.851 | 0.345 |
| CRP | 0.019 | 0.066 | 0.108 | 0.100 |
| TAC | 0.048 | 0.565 | 0.916 | 0.489 |
| MDA | 0.072 | 0.496 | 0.197 | 0.968 |
| SAA | 0.069 | 0.080 | 0.105 | 0.080 |

Interleukin-6 (IL-6); C-reactive protein (CRP); Serum amyloid A (SAA); Malondialdehyde (MDA); Total antioxidant capacity (TAC). Statistical significance was set at p < 0.05
